# Comparative Genomic and Phylogenetic Analysis of *Salmonella enterica* Isolated From Donkey

**DOI:** 10.1155/tbed/3032324

**Published:** 2025-11-13

**Authors:** Yuhui Tian, Xiaomeng Chen, Yan Su, Xinyu Jiang, Lingling Su, Baojiang Zhang, Jingxuan Peng

**Affiliations:** ^1^Department of Microbiology and Immunology, College of Veterinary Medicine, Xinjiang Agricultural University, Urumqi, China; ^2^Xinjiang Key Laboratory of New Drug Research and Development for Herbivorous Animals, College of Veterinary Medicine, Xinjiang Agricultural University, Urumqi, China; ^3^Feed Research Institute, Xinjiang Academy of Animal Science, Urumqi, China

## Abstract

*Salmonella* is one of the most prevalent foodborne and zoonotic pathogens threatening global public health and food safety. *Salmonella enterica* subspecies *enterica* serovar Abortusequi (*S*. Abortusequi) is frequently reported as the etiological agent of equine abortion, septicemia, and polyarthritis. Currently, comprehensive studies on the virulence traits, evolutionary dynamics, and genomic diversity of donkey *S*. Abortusequi are limited. In this study, we assessed the genomic epidemiology, antibiotic resistance, and pathogenic traits of a ST251 *S*. Abortusequi strain (XJP1), isolated from donkey, using the core genome single nucleotide polymorphism (cgSNP) phylogenetic analysis, core genome multilocus sequence typing (cgMLST), comparative genomic analysis, the antimicrobial susceptibility tests, and virulence assays. Our cgSNP and cgMLST analyses indicate that XJP1 is closely related to *Salmonella enterica* subspecies *enterica* strains isolated from humans, equines, donkeys, poultry, and associated with multiple serovars. Additionally, XJP1 exhibited a multidrug-resistant profile, showing resistance to seven antibiotics. In a mouse infection model, XJP1 showed significantly higher lethality compared to the XJS1 strain. Notably, XJP1 harbored a plasmid containing an IncFIB/IncFII incompatibility group replicon and carrying the *spvBC* operon, as well as the *rck*, *TTSS*, *pefABCD*, *fdeC* virulence genes, and *gadX* acid resistance transcriptional activators gene. Our findings suggest that this plasmid may be a critical virulence determinant in XJP1. In conclusion, our data on the epidemiology and pathogenicity of ST251 *S*. Abortusequi provide valuable insights into the genetic, virulence, and transmission dynamics of donkey *Salmonella enterica*, highlighting the need for attention to food safety measures and public health surveillance.

## 1. Introduction

*Salmonella enterica* is one of the most common pathogens causing zoonotic and foodborne illnesses, characterized by a wide host range and significant economic costs and public health concerns worldwide [1]. It ranks as the leading cause of foodborne bacterial diseases in China and the USA [2, 3] and has been listed by The World Health Organization (WHO) as one of the serious global health concerns. As of now, a total of 2695 *Salmonella* serovars have been identified [4], with the majority capable of infecting a broader range of hosts and are associated with major livestock and human diseases [5]. *S*. *enterica* subspecies *enterica* serovar Abortusequi (*S*. Abortusequi) is a frequently reported pathogen of equines and is classified as a nontyphoidal *salmonella* (NTS), which is considered a priority for global surveillance. *S*. Abortusequi was first reported in the USA in 1893 and has since been detected in both horses and donkeys in Italy [6], Argentina [7], Croatia [8], India [9], Russia [10], and Japan [11]. In China, the *S*. Abortusequi infection in horses and donkeys is associated with a high abortion rate, ranging from 30% to 100%, and is widespread among farms in Xinjiang, Shandong, Inner Mongolia, Hebei, and Heilongjiang provinces in China [12].

Although most *S*. Abortusequi infections are typically subclinical, acute abortions can occur during late pregnancy. Moreover, infected mares could be carriers and disseminators of pathogen. This *Salmonella* pathogen has also been considered an important causative agent of food poisoning, gastroenteritis, paratyphoid, and typhoid-like illness [11]. In some cases, the infection has resulted in laminitis, polyarthritis, and pneumonia. If let untreated, the infection can progress to neonatal septicemia and death [6, 13]. In recent years, equine abortion caused by *S*. Abortusequi has been effectively controlled in the United States, Argentina, and Europe and with only sporadic cases reported. In contrast, the number of abortions has increased in Africa and Asia [12, 14, 15]. China has the largest donkey population globally, with a booming donkey husbandry industry [12]. Recently, a complete donkey industry chain has emerged, significantly contributing to the country's economy [16]. Donkey meat, milk, and erjiao are becoming increasingly popular due to their nutritional value, unique flavor, and special functions. Despites the crucial economic impact, a high prevalence of *S*. Abortusequi in donkeys has been reported in the last decade. Consequently, the food safety of donkey meat has gained increasing attention. The high abortion rate caused by *S*. Abortusequi has also seriously hampered the development of donkey husbandry [14, 17]. However, in-depth investigation into the resistance, pathogenic mechanisms, genetic features, and transmission dynamics of *S*. Abortusequi in different hosts are lacking.

Multidrug-resistant *Salmonella* often results from the modification and transfer of antibiotic resistance-related genes through horizontal gene transfer, allowing spread among humans, animals, and the environment [18]. However, the exact mechanisms underlying this proliferation remain unclear. Compared to traditional microbiological or serological tests, advanced bioinformatics and whole genome sequencing (WGS) technology provides faster monitoring and more accurate results. These methods can be employed to characterize the genetic features and resistance patterns of *S*. Abortusequi. They can also be used to clone and determine the distribution of strains in various hosts, offering higher-resolution genetic clustering and enabling more precise identification of outbreak source.

Molecular methods, such as variable number of tandem repeat analysis and multilocus sequence typing (MLST), have been used to distinguish between *Brucella* strains [19, 20]. Furthermore, WGS-based phylogeny represents the most powerful tool for obtaining more genetic features and facilitating epidemiological investigation through phylogenetic analysis. WGS and MLST have been applied to characterize and clone *S. enterica* and determine its distribution in various hosts and environments [21].

This study was conducted due to limited research on NTS *S*. Abortusequi infections in donkey and their potential risks to public health. We aimed to investigate the genetic characteristics and evolutionary relationships of XJP1 molecular typing, virulence factors (VFs) and antibiotic resistance-related genes. Core genome single nucleotide polymorphism (cgSNP) and core genome MLST (cgMLST) were performed. Additionally, we investigated its pathogenicity by analyzing VFs, antibiotic resistance genes, and phenotypic antimicrobial resistance profiles. The data from this study contribute to a comprehensive understanding of the pathogenic and drug resistance-related molecular mechanisms of XJP1, as well as its genetic characteristics and evolutionary relationship, providing information crucial for tracing the source, prevention, and treatment of the disease in China.

## 2. Materials and Methods

### 2.1. Strain and Growth Conditions

XJP1 was isolated from the livers of diseased donkeys during a severe disease outbreak in Xinjiang, China, characterized by newborn deaths at birth and in female donkey abortions. It was identified as *S*. Abortusequi based on its phenotype on XLD agar plate (Qingdao Hope Bio-Tcehnology Co., Ltd., China). Positive cultures were further characterized by Gram staining, biochemical tests, 16S rRNA sequencing, and PCR assays. In our study, pure cultures of *Salmonella* were subjected to laboratory testing, with nine isolates confirmed as *S*. Abortusequi and shown to be of the same sequence type, ST251. The XJP1 strain and reference control strain *S*. Abortusequi XJS1 (a horse S. Abortusequi isolate, preserved in our lab) were cultured overnight in Luria–Bertani (LB) nutrient broth at 37°C.

### 2.2. DNA Extraction, WGS, Assembly, and Annotation

Genomic DNA was extracted from the overnight XJP1 culture incubated at 37°C using a TIANamp bacteria DNA kit (Tiangen, Beijing, China) following the manufacturer's instructions. The DNA concentration and quality were determined using a NanoDrop 1000 spectrophotometer (Thermo Fisher Scientific, USA) and 0.8% agarose gel electrophoresis. Briefly, WGS was carried out using both the Illumina NovaSeq platform (Illumina, San Diego, CA, United States) and the long-read PacBio RS II platform (Pacific Biosciences, CA, United States). De novo assembly of reads was acquired by using the continuous long reads and according to the Hierarchical Genome Assembly Process workflow (Pacific Biosciences) using the SMRT Analysis System v2.3.014. The genome coverage was 43.8×. All processed reads were assembled using SOAP denovo v2.04. Final annotations were performed using the NCBI prokaryotic genome annotation pipeline (PGAP) v6.8. The open reading frame finder (http://www.ncbi.nlm.nih.gov/orffinder) identified the features of XJP1. Genome annotation was performed by employing RAST (http://rast.nmpdr.org) and BLASTn (http://blast.ncbi.nlm.nih.gov/Blast.cgi).

### 2.3. Phylogenetic Analysis

For phylogenetic analysis, 81 assembled genome sequences of different serovars of *S. enterica* strains were downloaded from the NCBI database. The phylogenetic relationship of these strains was performed using snippy v4.6.0 (https://github.com/tseemann/snippy) and recombination was excluded from the alignment. Tree calculation was performed using IQ-Treev.3.0.1 [22]. The PubMLST database (https://pubmlst.org/) was utilized for cgMLST prediction of the 133 *Salmonella* strains from 15 serovars.

### 2.4. Sequence Analysis and Virulence Gene Identification

Genomic islands (GIs) were identified by employing IslandViewer 4 [23]. Integrative conjugative elements were predicted using ICEfinder (https://bioinfo-mml.sjtu.edu.cn/ICEfinder). Plasmid Finder-1.3 (https://cge.cbs.dtu.dk/services/PlasmidFinder/) was applied to identify the plasmid replicon types [24]. Based on the phylogenomic analysis results, eight *S*. Abortusequi strains were selected for genome comparison with XJP1. The circular chromosomes of these nine strains were visualized utilizing the BLAST Ring Image Generator (BRIG) v0.95 [25], with all identified GIs and prophages of XJP1 marked on the outermost ring. Furthermore, the circular plasmids were aligned and visualized using BRIG v0.95, employing the pXJP1 plasmid as the reference. The antimicrobial resistance-related genes of XJP1 were annotated using the Comprehensive Antibiotic Resistance Database (CARD; https://card.mcmaster.ca/analyze). VF-encoding genes within the XJP1 genome were predicted utilizing the VF database (VFDB; http://www.mgc.ac.cn/VFs/main.htm) [26].

### 2.5. Antimicrobial Susceptibility Testing

XJP1 was subjected to antimicrobial susceptibility testing using the Kirby–Bauer disk diffusion method per the Clinical and Laboratory Standards Institute guidelines [27]. Testing was performed utilizing the eight antibiotics: ampicillin, cefuroxime, doxycycline, tetracycline, levofloxacin, ciprofloxacin, and sulfamethoxazole. *Staphylococcus aureus* ATCC 25,923 was employed as a quality control strain.

### 2.6. Pathogenicity Testing in Animal Models

In vivo challenge experiments were performed using the mice model. In brief, XJP1 and XJS1 (control group) was cultivated, precipitated, washed, and resuspended in phosphate-buffered saline. 18 Female BALB/c mice (six per group; age 6–8 weeks) were randomly divided into three groups and intraperitoneally injected with XJP1 or XJS1 solution (2.5 × 10^7^ CFU); PBS was used as a negative control. Then, the mortality of mice was observed and recorded for 24 h. Dead mice were necropsied at the time of death, whereas surviving animals from each group were anesthetized with xylazine (9 mg/kg) and ketamine (36 mg/kg) and euthanized via cervical dislocation 24 h post-infection. Statistical analysis of the data was conducted employing GraphPad Prism, with survival data displayed using the Kaplan–Meier method.

To comprehensively assess the pathogenicity of XJP1 and XJS1, livers and spleens from the infected mice were aseptically collected 24 h post-infection. Bacterial load enumeration involved homogenization and lysis of the organs with PBS buffer. The lysates were serially diluted and plated onto LB agar. The resulting colonies were then enumerated after overnight incubation at 37°C. The impact of XJP1 or XJS1 infection was evaluated by histopathological examinations of the livers and spleens (*n* = 3 of each organ) after intraperitoneal challenge with 2 LD_50_ (2.5 × 10^7^ CFU). The excised organs were fixed in 4% paraformaldehyde and stained with hematoxylin (HE).

### 2.7. Statistical Analysis

Statistical analysis was conducted using SPSS software version 10.0 (IBM, NY, USA). Mice survival rates (%) and other numerical data are presented as the mean ± SD. After performing the arcsine square root conversion of the survival rates, these data were subjected to one-way variance analysis to determine the statistical significance (*p*  < 0.05), followed by Tukey's post hoc test. ns *p*  > 0.05 indicates no significant differences, *⁣*^*∗*^*p*  < 0.05 indicates significant differences, *⁣*^*∗∗*^*p*  < 0.01 indicates highly significant differences, and *⁣*^*∗∗∗*^*p*  < 0.005 or *⁣*^*∗∗∗∗*^*p*  < 0.001 indicates extremely significant differences.

## 3. Results

### 3.1. Features of the XJP1 Genome

The XJP1 genome consists of a single circular chromosome and circular plasmid (Table 1). In total, the chromosome comprised of 4,739,554 bp with 4639 predicted protein-coding sequences (CDSs), along with 75 tRNAs, 22 rRNAs, and 200 sRNAs. The average GC content was 52.2%. The plasmid was identified and designated as pXJP, with a total size of pXJP1 was 93,802 bp, and the average GC content was 53%. Additionally, two CRISPR-cas sequences were also identified.

### 3.2. Phylogenetic and MLST Analysis

**Figure 1:**
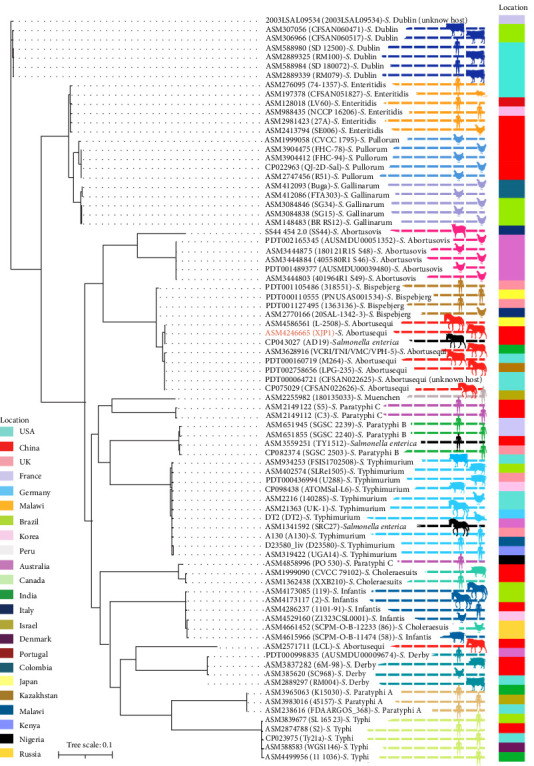
Phylogenetic tree based on core genome SNPs. The phylogenetic relationship of XJP1 with 80 other serovars was assessed. *Salmonella* genomes were downloaded from the GenBank, and the phylogenetic tree was constructed using Snippy v.4.6.0. Different colors were assigned to various serovars and black for unknown. The source and country are displayed on the right side of the map. All sequences are indicated by their assembly names.

The phylogeny of XJP1 was compared with 80 other *Salmonella* strains based on their core and accessory genomes. As shown in Figure 1, XJP1 clustered closely with the donkey-isolated strains CP043027(AD19) (China), ASM3628916 (India), PDT000160719 (USA), and two horse-derived strains ASM4586561 (Japan) and PDT002758656 (Kazakhstan), indicating high genetic similarity. Of these, XJP1 and CP043027 (AD19) originated from China. Notably, seven *S*. Abortusequi strains, one avian *S*. Bispebjerg isolate ASM2770166, and three human *S*. Bispebjerg strains (PDT001105486, PDT000110555, and Ptd001127495) were grouped within the same clade, indicating a relatively close relationship among these seven donkey *S*. Abortusequi strains and the human and avian *S*. Bispebjerg strains. In another clade, one donkey-derived *S*. Abortusequi strain ASM2571711 (China) clustered closely with one human *S*. Derby strain PDT000998835 (Australia), one swine *S*. Derby isolate ASM3837282 (China), one avian *S*. Derby isolate ASM385620 (China) and one bovine *S*. Derby isolate ASM2889297(USA), indicating a relatively close relationship between the donkey *S*. Abortusequi strain ASM2571711 (China) and human, avian, swine and bovine *S*. Derby strains.

**Figure 2:**
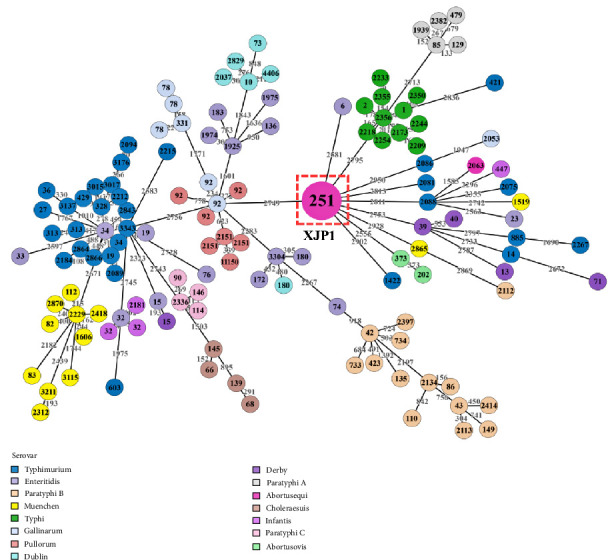
Minimum Spanning Tree (MST) based on the cgMLST analysis of the sequence types of 133 *Salmonella* strains of 15 serovars. MST allowed the visualization of the number of loci variations between these genomes. Numbers by lines represent the number of strain-specific loci. The numbers in each circle represent the sequence type (ST) of these strains, and the diameter of each circle shows the number of strains present. The ST type of XJP1 was 251 and was indicated with the red dashed box.

Based on the pubMLST database, XJP1 was calssified as ST251. A minimum spanning tree (MST) was constructed using the sequence types of 133 *Salmonella* strains from 15 serovars. This showed the divergent evolution of XJP1 from China in relation to other *S. enterica* strains of different serovars from other countries and sources. The XJP1 (ST251) was linked to the *S. enterica* serovars Typhimurium, enteritidis, Derby, Typhi, paratyphi A, Gallinarum, Abortusovis, Infantis, and Muenchen (Figure 2).

### 3.3. Genome and Plasmid Comparisons

**Figure 3:**
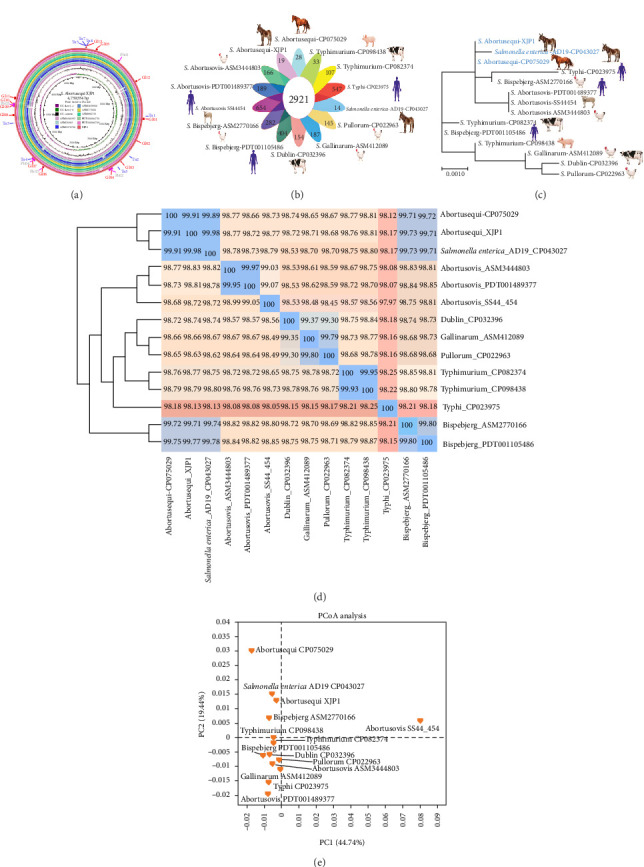
Sequence alignment and comparative genomic analysis of XJP1 with other *Salmonella enterica* strains from different host. (a) Comparison of the eight closed circular genomes of *S*. Abortusequi strains in reference to the genome of the *S*. Abortusequi XJP1 strain using BRIG. Query genomes are plotted from the innermost to the outermost ring. (b) Venn diagram showing the number of shared and unique orthologous genes among XJP1 and 13 *Salmonella enterica* strains of different host and serovars. (c) A phylogenomic tree was constructed based on 16S rRNA genes of six related *S*. e*nterica* reference strains. (d) Heatmap based on average nucleotide identity (ANI) values between each pair of genome sequences from strain *S*. Abortusequi XJP1 and 13 related *S*. *enterica* strains. (e) Principal component analysis based on COG function of the genomes from XJP1 and 13 *S*. *enterica* reference strains.

The chromosome of XJP1 was compared with those of *Salmonella* strains isolated from horses and donkeys (Figure 3a). Overall, these nine strains shared high nucleotide similarity; however, some variable regions also existed, particularly in ASM1341592 (horse strain), ASM21363 (horse strain), and ASM2571711 (donkey strain). Among the 13 GIs carried by XJP1, GI-8 was the largest, with a size of 59.7 kb.

The genomes of 14 *Salmonella* isolates: AD19-CP043027 (donkey isolate), Abortusequi-CP075029 (horse isolate), Dublin-CP032396 (bovine isolate), Typhi-CP023975 (human isolate), Bispebjerg-ASM2770166 (avian isolate), Abortu-sovis-PDT001489377 (human isolate), Abortusovis-SS444542 (sheep isolate), Abortusovis-ASM3444803 (avian isolate), Typhimurium-CP098438 (swine isolate), Typhimurium-CP082374 (bovine isolate), Bispebjerg-PDT001105486 (human isolate), Gallimarum-ASM412089 (avian isolate), Dublin-CP032396 (bovine isolate), and Pullorum-CP022963 were aligned and compared to the XJP1 genome to ascertain the number of shared and unique orthologous genes, phylogenomic relationship based on 16S rDNA, average nucleotide identity (ANI), and cluster of orthologous group (COG) categories. Among these, three human isolates, Abortusovis-SS44454 (654), Typhi-CP023975 (547), and Bispebjerg-PDT001105486 (404) had more unique genes (Figure 3b). The phylogenetic tree suggested that XJP1 was closely related to AD19-CP043027 (Figure 3c) and Abortusequi-CP075029, which is consistent with the ANI (Figure 3d) and PCoA (Figure 3e) results.

**Figure 4:**
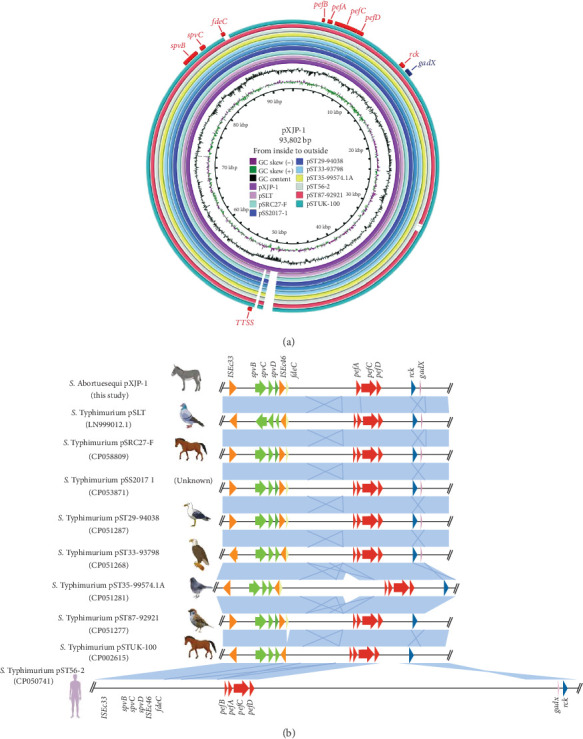
Circular map and comparative analysis of t plasmid pXJP1. (a) In this study, pXJP1 was used as a reference and compared with nine *Salmonella enterica* plasmids utilizing BRIG. The deep blue circle represents the pXJP1 plasmid. From the center: pXJP-1 (donkeys), DT2 (pigeons), pSTUK-100 (horses), pSRC27-F (horses), pSS2017-1 (unknown), pST29-94,038 (gulls), pST35-99574.1A (juncos), pST87-92,921 (sparrows), and pST56-2 (*Homo sapiens*). (b) Schematic representation and linear pairwise comparison of plasmid pXJP1 with other similar plasmids of *Salmonella enterica* from different host. Resistance genes, mobile elements, and virulence genes are denoted by arrows. Shading area denotes the regions that exhibit genetic similarity among different plasmids.

Isolate XJP1 harbored a single plasmid (93,802 bp) that contained nine VF-encoding genes, *pefA*, *pefB*, *pefC*, *pefD*, *spvB*, *spvC*, *Rck*, *TTSS*, and *fdeC* were detected. BLAST analysis revealed that, with the exception of *FdeC*, the other nine genes had homologs on the plasmids of some *S*. *enterica* strains isolated from different host (Figure 4a and Table 2). Additionally, we retrieved nine plasmids of the global *Salmonella* isolates from NCBI for collinearity analysis. The multireplicon plasmids that carried in XJP1 and other nine isolates comprised both IncFIB and IncFII types. This multireplicon plasmid exhibited a wide host range, including horse, donkey, *homo sapiens*, pigeon, junco, sparrow, and hawk (Table 2).

Collinearity relations and structural variations are visualized in Figure 4b. Notably, pXJP1 was closely related to plasmids of *Salmonella* strains isolated from horses, donkeys, pigeons, gulls, juncos, sparrows, hawks, and humans. Furthermore, pXJP1 and these plasmids contained the acid-resistant transcriptional activator-encoding gene *gadX* (Figure 4a,b).

### 3.4. Compare Genome and the Pathogenic Factors of XJP1

**Figure 5:**
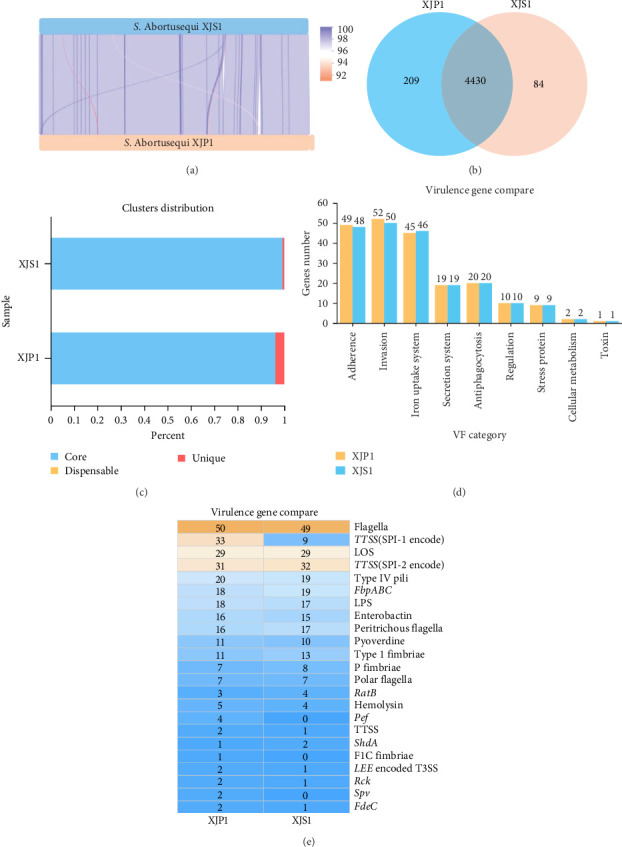
Comparison of the genomes of XJP1 and XJS1 (a) Collinear alignment of the genomes of XJP1 and the reference *S*. Abortusequi strain XJS1. (b) Venn diagram of the unique and consensus genes in XJP1 and XJS1. (c) Cluster distribution of core, dispensable and unique genes of XJP1 and XJS1. (d) Comparison of the different classes of putative virulence genes of XJP1 and XJS1. (e) Comparison and distribution of the different virulence genes in the *S*. Abortusequi isolates XJP1 and XJS1.

Genome synteny was further explored by comparing the linear organization of the chromosomes of XJP1 and reference control strain *S*. Abortusequi XJS1. As shown in Figure 5a, a high degree of global synteny was observed between the two isolates from China, XJP1 and XJS1. Some genome inversions and rearrangements were identified between the donkey strain XJP1 and XJS1. Additionally, XJP1 posessed more unique genes (209) compared to XJS1 (84) (Figure 5b,c). To illustrate the pathogenic potential of XJP1 strains, we scanned the virulence related factors in the genome of XJP1 and the reference control strain XJS1. The VFs were retrieved by querying the XJP1 genome in the VFDB database. The functions of these VFs primarily include secretion, iron uptake, adherence, antiphagocytosis, invasion, toxin, serum resistance, magnesium uptake, stress proteins, and phase variation (Figure 5d and Table 3). XJP1 possessed a great number of adherence-associated (*n* = 49) and invasion-associated virulence genes (*n* = 52) but fewer iron uptake system-associated VFs (*n* = 45) compared to the *S*. Abortusequi-XJS1. Furthermore, XJP1 contained additional virulence-related genes, including TTSS, type Ⅳ Pili, T3SS, Pef, fdeC, rck, Spv, LPS, enterobacin, hemolysin, flagella, and pyoverdine compared to XJS1 (Figure 5e).

### 3.5. Antimicrobial Resistance-Encoding Genes

The CARD database was employed to denote the resistance-related genotypes on the genome of XJP1. The results indicated that 242 antimicrobial resistance-associated genes of XJP1 were identified in XJP1 (Table 4). Among these, 241 were harbored on the chromosome, and only one was found on the plasmid. According to the CARD database, XJP1 contains 33 quinolone resistance-associated genes, including *oqxA*, *efrA*, *patA*, and others.

### 3.6. Antimicrobial Susceptibility Profiles

We subsequently assessed the resistance phenotypes of XJP1. Antimicrobial susceptibility testing revealed that the XJP1 exhibited resistance to seven antibiotics, including cefuroxime, doxycycline, tetracycline, levofloxacin, ciprofloxacin, and sulfamethoxazole (Table 4). Consequently, XJP1 displayed a multiple drug resistance profile.

### 3.7. Virulence-Associated Phenotypes of XJP1

**Figure 6:**
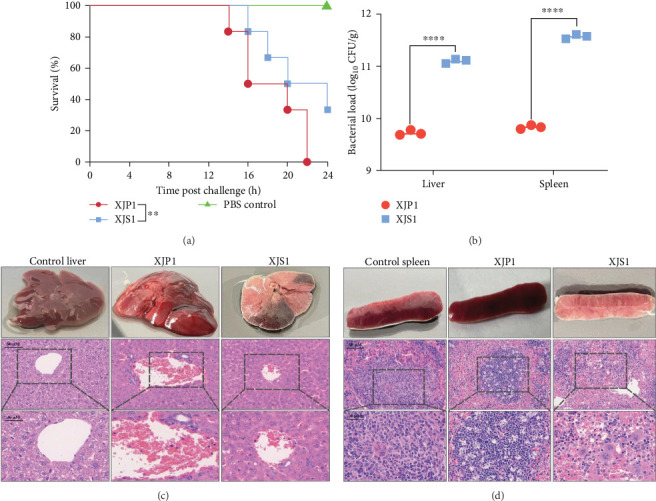
Virulence analysis of XJP1 compared with XJS1. (a) Survival analyses of mice (*n* = 6 per group), which were infected intraperitoneally with XJP1 and XJS1 (2.5 × 10^7^ CFU) and monitored daily for morbidity and mortality for 24 h. (b) Bacterial load (CFU/g) in the livers and spleens of mice (*n* = 3) challenged with XJP1 and XJS1 (2.5 × 10^7^ CFU) was determined. All data are presented as the mean ± SD. ns *p* > 0.05, *⁣*^*∗*^*p* < 0.05, *⁣*^*∗∗*^*p* < 0.01, *⁣*^*∗∗∗*^*p* < 0.005, and *⁣*^*∗∗∗∗*^*p* < 0.001. (c) Representative histopathological H&E images (scale bar = 30 or 60 μm) of livers, and their sections extracted from mice challenged intraperitoneally with XJP1 (5 × 10^7^ CFU). (d) Representative histopathological H&E images (scale bar = 30 or 60 μm) of spleens, and their sections extracted from mice challenged intraperitoneally with XJP1 (5 × 10^7^ CFU).

In the mouse infection model, the negative control group mice retained asymptomatic and in good health. Compared to the control group, mice infected with XJP1 and XJS1 exhibited symptoms, such as subdued behavior, decreased appetite, and lethargy during the observation period. As shown in (Figure 6a), the percentage of surviving mice infected with XJS1 (30% survival at 24 h) was significantly higher than that of mice infected with XJP1 (0% survival at 22 h, *p*  < 0.05), suggesting that the XJP1 isolate is more virulent than control strain XJS1.

In the mouse infection model, both the *Salmonella* load in the livers and spleens of mice infected with XJP1 was significantly lower than that in mice infected with XJS1 (*p*  < 0.01), indicating that XJP1 possesses high virulence capacity, as fewer XJP1 can cause higher mortality in mice (Figure 6b). Based on the analyses and comparison of VFs and plasmid of XJP1, we speculate that the virulence genes present only on the plasmid of XJP1 might be responsible for the higher pathogenicity.

Additionally, we evaluated tissue injury and inflammatory cell infiltration by H&E staining. The representative microscopy images of the liver and spleen tissues from mice 24 h post-infection with XJP1, XJS1 and unchallenged control are shown in Figure 6c,d). Mice infected with XJP1 exhibited severe liver damage, characterized by prominent inflammatory infiltration and necrosis of multiple hepatocytes. The splenic architecture was disorganized histologically, with indistinct demarcation between the cortex and medulla, as well as a disorganized medullary region.

## 4. Discussion

*Salmonella* is a multihost and widely distributed food pathogen [28]. In China, Salmonellosis accounts for around 70%–80% of bacterial foodborne diseases, with meat products were identified as the primary contaminated food source and exhibiting the highest variety of *salmonella* serovars [29]. Humans typically acquire NTS infections by consuming poultry and livestock meat. According to previous studies, the threat of NTS infection is observed to be higher in northern China than in southern China [30], suggesting that greater attention should be paid to food animal health, food processing procedures, and the need for advanced surveillance. While sporadically reported in the USA and Europe, *S*. Abortusequi is commonly isolated from Asia and Africa [31, 32]. In Japan, *S*. Abortusequi has been listed as a notifiable disease due to its high infectivity and adverse economic impact [33]. Equine abortus salmonellosis in horses and donkeys has occurred frequently in recent years in China. However, reports of donkey infections caused by *S*. Abortusequi are limited and not thoroughly investigated [12, 17].

Advances in WGS of *salmonella* over the last decade, which utilize data from the entire genome, have allowed for a better understanding of the underlying genetic determinants of antimicrobial-resistant and the epidemiological characteristics of *S*. Abortusequi. In recent years, several population genomic studies of *S*. Enteritidis have been conducted [34], but genetic characterization and epidemiological analyses of *S*. Abortusequi are lacking. In this study, a donkey *S*. Abortusequi isolate, XJP1, was typed as ST251, which has been previously reported for isolates from Croatia, Argentina, Italy, and the United States [6]. Therefore, we characterized *S*. Abortusequi XJP1 isolated from Chinese donkeys, along with other *S*. *enterica* ssp. *enterica* isolates from the public database. Additionally, we performed an extensive epidemiological study of this disease to monitor and understand its transmission routes.

Considering the zoonotic nature of *Salmonella*, examining the genetic relatedness between isolates is of great significance. Genomic SNP and MLST analysis provide insights into genetic diversity and relatedness. In the present study, we retrieved an additional 80 genomes of global *Salmonella* isolates from the NCBI to investigate the genetic relationships based on the core genome. Phylogenetic reconstruction divided these isolates into 15 clades. Notably, in one clade, four donkey-derived *S*. Abortusequi strains, including the Chinese isolate XJP1(ASM4246665), Chinese isolate AD19 (CP043027), the USA strain (PDT000160719), the Indian strain (ASM3628916), and two horse-derived *S*. Abortusequi strains, the Japanese strain (ASM458656), and the Kazakhstan strain (PDT002758656) have a close relationship with one poultry-derived strain and three human derived *S*. Bispebjerg isolates. Furthermore, another donkey-derived *S*. Abortusequi strain ASM2571711 (China) was closely related to four human, avian, swine, and bovine-derived *S*. Derby isolates. This finding indicates that *S*. Abortusequi donkey strains have close relationship with *S*. Bispebjerg and *S*. Derby strains from various sources. *S*. Bispebjerg is a rare serovar, first reported in turkey in 2011 [35] and subsequently detected in human cases (2016) and food (2013). Additionally, *S*. Derby is a swine-adapted serovar that causes foodborne disease outbreaks worldwide through contaminated pork and poultry products. It is rarely detected in humans [36].

Globally, the most commonly reported serovars in animal-derived foods and human cases are *S*. Typhimurium, *S*. Enteritidis, and the emergent *S*. Infantis [28, 37]. In China, *S*. Typhimurium, *S*. Enteritidis, *S*. Derby, and *S*. Agona are serovars reported in animal-derived foods and human case [30]. Consistent with this, our phylogenomic analysis revealed that *S*. Typhimurium and *S*. Infantis strains, capable of infecting equine and another China donkey *S*. Abortusequi strain ASM2571711 is closely linked with the *S*. Derby strain. This correlation may have public health implications.

In this study, we retrieved an additional nine plasmids from global *Salmonella* isolates in the NCBI and compared them with pXJP1. Notably, pXJP1 was very closely related to plasmids of *Salmonella* strains isolated from horses, donkeys, pigeons, gulls, juncos, sparrows, hawks, and humans. This result aligns with the findings from the XJP1 cgSNP and cgMLST analyses. Chickens have long been recognized as important reservoirs for *Salmonella* [38], with the potential to transmit antibiotic-resistance genes to humans through the food chain, thus, posing a severe threat to public health [39].

cgSNP analysis and plasmid comparison not only offered insights into the genetic relatedness between XJP1 and *S. enterica* isolates but also suggested the potential zoonotic transmission of *Salmonella* among humans, donkeys, and poultry, indicating that the donkey-derived *S*. Abortusequi, is associated with poultry *Salmonella*. This finding aligns with a previous study reporting a closely relationship between a plasmid from a poultry-associated strain and a plasmid from *E. coli* detected on a sheep farm in the United Kingdom [40].

Next, we examined the relationship of ST251(XJP1) to other serovars using cgMLST, a well-established molecular typing method characterized by low levels of missing data and sensitivity comparable to core SNP typing. This method has been used successfully used for the structural determination of bacterial populations and for tracing disease transmission chains. *Salmonella*-caused diseases in humans are associated with isolates from farms or food products [41] and can infect humans through the food chain [42]. MLST analysis, based on the sequence types of the 133 *Salmonella* strains from 15 serovars revealed that the clustering of isolates exhibited a high degree of agreement with the genome SNP-based analysis. The MLST data indicated that the donkey strain XJP1 (ST251) has a close association with *Salmonella* serovar Typhimurium, Typhi, Enteritidis, Paratyphi A, Gallinarum, Abortusovis, and Muenchen, isolated from various animal and human sources. Notably, this data further supports the findings of the cgSNP analyses, which also indicated that XJP1 was closely related to the poultry-derived *S*. Gallinarum (ST92) and *S*. Abortusovis (ST373).

*S*. Abortusovis is a sheep-adapted pathogen that causes spontaneous abortion [43]. It was detected in poultry in Australia in 2009, and more recently, human infections due to poultry-associated *S*. Abortusovis have been reported [44]. Epidemiological results indicated that this pathogen is widely distributed in New South Wales poultry and has caused sporadic human infections. Animals that serve as food, particularly chickens, are long-recognized important reservoirs for *Salmonella* [38], making the contamination of poultry by *S*. *enterica* a major route of transmission to humans.

WGS and MLST can be employed to characterize pathogens, clone their genomes, and determine their distribution in various environments and hosts [21]. In the present MLST analysis, XJP1 clustered with the isolates ST373, ST6, ST92, ST2086, ST2356, and ST2865 obtained from humans, animals, food, or farms. Among these isolates, *S*. Enteritidis (ST6) and *S*. Typhi A (ST2356) were derived from clinical samples, while *S*. Typhimurium (ST2086) and *S*. Muenchen (ST2865) were isolated from animals, food, or environment. These results suggested potential transmission of *Salmonella* among humans, animals, food, and the environment. The cgMLST analysis of XJP1 (ST251) identified slightly more distantly related isolates of ST6, which may represent longer transmission chains.

Plasmids can encode virulence- and drug resistance-related genes, promote bacterial diversity, and facilitate adaptation through horizontal gene transfer. A typical plasmid normally contains one replicon, but multireplication plasmids that carry two or more replicons have been frequently reported. In XJP1, IncFIB(S) and IncFII(S) multireplicon incompatibility types were identified. The multireplicon status may allow a plasmid to have a broad host range for replication. Since IncFIB/IncFII plasmid can carry both antimicrobial resistance genes and various VFs, their spread among foodborne pathogens is a significant public health concern. These plasmids are frequently found in *E. coli* and *Salmonella*, serving as reservoirs that facilitate plasmid transfer between Gram-negative bacteria [45, 46].

They are the main plasmid replicons belonging to the IncF family and are widely distributed in Enterobacteriaceae, especially *S. enteritidis* and *E. coli*. IncF plasmid replicons promote bacterial infection and drug resistance [47]. The presence of virulence-related genes on plasmids enhances bacterial colonization and adhesion to host cells. In this study, the *spvB* and *spvC* genes were identified as relating to plasmid transmission. *Spv* operon suppresses the host's innate immunity [48, 49]. Using a mouse systemic infection model, *spv* was identified as an essential VF [50]. *SpvB* disrupts the integrity of intestinal epithelial cells, increasing permeability and supporting *Salmonella* translocation [51]. *spvC* inhibits the autophagy of host cells [52] and is relevant for the rapid growth and survival of *S*. Abortusequi within the host, proving essential to its virulence. Additionally, the plasmid contained virulence-related genes like *rck*, *fdeC*, *TTSS*, and *pefABCD* virulenc genes. *FdeC*, which was first identified as one of the *E. coli* VFs that bind to the host cells, has rarely been reported in *salmonella* infection. *FdeC* is located on the bacterial surface, with expression triggered by the interaction of *E. coli* with host cells. As an adhesin, *fdeC* contributes to colonization, an essential process for *S*. Abortusequi virulence and infection. Furthermore, *fdeC* has been shown to significantly contribut to biofilm formation of avian pathogenic *Escherichia coli* [53]. To further assess the virulence potential of XJP1, we established mouse infection models. Infection with XJP1 caused significantly greater mortality compared to the control group XJS1 which lacks plasmid. Additionally, the bacteria load of the XJP1 isolate was significantly lower than that of XJS1.

Consistent with the antimicrobial resistance rates, the ARG results indicated that XJP1 possessed not only the chromosome-mediated but also the plasmid-mediated gene (*gadX*). Previous research demonstrated that the *E*. *coli* regulator *gadX* plays a critical role in the transcriptional regulation of the glutamate-dependent acid resistance system. The *E*. *coli gadX* mutant exhibited a wide range of effects on the transcription of various genes at pH 5.5 when compared to the wild-type [54]. GadX regulates several genes in enteropathogenic *E*. *coli* [55–57]. Recently, *gadX* was reported to indirectly regulate *fim* transcription [58]. In this study, the acid-resistant transcriptional activator-encoding gene (*gadX*) was found on pXJP1, potentially enabling survival in acidic environments.

The transmission of antibiotic-resistant *Salmonella* between humans and animals is a major concern for both human and animal health. The potential transmission of ampicillin-resistant *S*. *typhimurium* has been associated with the use of penicillin in animal feed [59]. The present study reports that XJP1 exhibited resistance to seven tested antibiotics, including quinolones (levofloxacin, ciprofloxacin, and norfloxacin), tetracyclines (doxycycline and tetracycline), sulfonamides (sulfamethoxazole), but sensitivity to β-lactams (ampicillin), indicating a broad antibiotic resistance profile. Fluoroquinolones (FQs) are frequently employed as a primary treatment for *Salmonella* infections in humans and animals [60, 61]. However, the misuse of FQs is leading to serious antibiotic resistance in both populations [60, 62] Nowadays, quinolone and β-lactam antibiotics are commonly used to treat *Salmonella* infections. FQs show relatively low biodegradability and are extensively used in several countries for the prevention and treatment of animal diseases [63]. Our results showed that XJP1 was resistant to three FQs (levofloxacin, ciprofloxacin, and norfloxacin). Of note, XJP1 carried multiple antimicrobial resistance-related genes on its chromosome and plasmid, which may contribute to FQ resistance.

We found that the XJP1 retained its susceptibility to one β-lactams (ampicillin), implying its potential effectiveness against abortion and diarrhea in donkeys. Thus, monitoring antimicrobial resistance in *S*. Abortusequi is necessary. Such measures may play a vital role in reducing the emergence and spread of antimicrobial resistance in both animals and humans.

This study has a few limitations. The number of *S*. *enterica* isolates of different serovars collected was not large enough, which may affect our comprehensive understanding of this lineage. Future investigation with larger sample sizes and more comprehensive genomic data would be beneficial for better understanding the *S*. *enterica* lineage and virulence traits.

## 5. Conclusions

To summarize, this is the first detailed and comprehensive genomic characterization of multidrug-resistant *S*. Abortusequi Chinese ST251 strains isolated from donkeys. Our cgSNP and cgMLST phylogenic and comparative genome analysis revealed that XJP1 was closely linked with poultry and human clades, suggesting potential animal reservoirs and transmission routes. Importantly, XJP1 possesses a large number of virulence and resistance determinants, as well as an IncFIB/IncFII plasmid containing the *spvBC* operon, the efflux pump-encoding gene *gadX*, as well as the *rck*, *TTSS*, *pefABCD*, and *fdeC* virulence genes, exhibiting pathogenicity in mice. Our research provides new insights into the epidemiology of *S*. Abortusequi ST251, highlighting the importance of initiating preventive measures and surveillance systems to safeguard donkey and public health while combating antibiotic resistance.

## Figures and Tables

**Table 1:** Genome annotation of XJP1 strain.

Features	XJP1 chromosome	XJP1 plasmid
Genome size (bp)	4,739,554	93,802
G + C content (%)	52.18	53.01
CDS number	4633	104
5S rRNA number	8	0
16S rRNA number	7	0
23S rRNA number	7	0
sRNA number	200	4
tRNA number	75	0
Prophages number	5	0
CRISPR regions number	2	0
Transposon number	6	0

**Table 2:** Antibiotic resistance, replicon types, and sizes of *Salmonella enterica* plasmids.

Plasmid name	Accession number	Bacteria	Source	Region	Year	Replicons	Size (kb)	ARGs	Isolate MLST
DT2-plasmid	LN999012	*S*. Typhimurium	Pigeon	Europe	2016	IncFIB(S)/IncFII(S)	9.3	—	ST128
pSRC27-F	CP058809	*Salmonella enterica*	Horse	Australia	2020	IncFIB(S)/IncFII(S)	9.3	—	ST19
pSTUK-100	CP002615	*S*. Typhimurium	Horse	America	2011	IncFIB(S)/IncFII(S)	9.3	—	ST19
pSS2017-1	CP053871	*S*. Typhimurium	Unknown	Belarus	2020	IncFIB(S)/IncFII(S)	9.5	—	ST19
pST29-94038	CP051287	*S*. Typhimurium	Gull	Canada	2020	IncFIB(S)/IncFII(S)	9.4	—	ST19
pST35-99574.1 A	CP051281	*S*. Typhimurium	Junco	Canada	2020	IncFIB(S)/IncFII(S)	9.9	—	ST19
pST87-92921	CP051277	*S*. Typhimurium	Sparrow	Canada	2020	IncFIB(S)/IncFII(S)	9.3	—	ST19
pST33-93798	CP051268	*S*. Typhimurium	Hawk	Canada	2020	IncFIB(S)/IncFII(S)	9.3	—	ST19
pST56-2	CP050741	*S*. Typhimurium	*Homo sapiens*	China	2011	IncFIB(S)/IncFII(S)	10.8	AAC(6′)-Il, sul1, AAC(3)-IId	ST7910
pXJP-1	—	*S*.Abortusequi	Donkey	China	2024	IncFIB(S)/IncFII(S)	9.3	—	ST251

**Table 3:** Virulence genes of XJP1.

Category	Class		Related genes
*Salmonella* pathogenicity islands (SPIs)	SPI-1	Iron, manganese transport	*sitABCD*
		T3SS (SPI-1 encode)	*sprB*, *hilACD*, *orgABC*, *iagB* *prgHKJI*, *sicAP*, *iacP*, *sipBCD*, *spaOPQRS*, *invABCEFGHIJ*
		T3SS-1 translocated effectors	*sptP*, *sipA*
	SPI-2	SPI-2 encoded T3SS	*ssrB*, *sscB*,
		T3SS (SPI-2 encode)	*ssrA*, *sseABCDE*, *ssaUTRQPOLKJHEDVMG*
		T3SS-2 translocated effectors	*sseGFIJLK1*
	SPI-3	MisL	*misL*
		Mg^2+^ transport	*mgtC*
	SPI-4	BapA	*siiE*
	SPI-5	T3SS-1 translocated effectors	*sopB*
		T3SS-2 translocated effectors	*pipB, pipB2*

Plasmid	Spv		*spvA*, *spvB*
	pef		*pefABCD*
	rck		*rck*

Adhesins	Type 1 fimbriae		*fimACDFHWYZ*
	Curli fibers, thin aggregative fimbriae (AGF)		*csgABCDEFG*
	Other adhesins		*lpfABCDE*, *stbBDE*, *stdBC*, *stfADF*, *sthADE*, *stiB*, *safBD*, *bcfABDEFG*, *pagC*, *pagN*

Flagella and chemotaxis	Peritrichous flagella		*FliRQPONMLKJIHGFETSDBAZY*, *fljB*, *flhABCDE*, *flgABCDEFGHIJKLMN*, *MotAB*, *cheABRWYZ*

Bacteriophage-encoded virulence factors	Enterobactin synthesis		*entABCDFS*
	LPS glucosylation		*gtrAB*
	T3SS-1 translocated effectors		*sopADD2E*, *sspH2*

Fimbriae	Type IV pili		*pilT*
	Other fimbriae		*steAC*

Lipopolysaccharide (LPS)	LPS		*fabZ*, *waaPG*
	LPS-modifying enzyme		*pagP*, *wecA*, *lpxDH*, *msbA*, *kdtA*, *rfaCF*
	Phosphoethanolamine modification		*lptA*

Capsule	Capsule		*oppF*, *wcaIH*, *gmd*, *wzb*
	Capsular polysaccharide		*wecB*

**Table 4:** Antimicrobial resistance phenotype and antimicrobial resistance genes of XJP1.

Class	Antimicrobial Agent	Resistance phenotype	Antimicrobial resistance genes
β-lactams	Ampicillin	S	*LRA-8*, *OmpK37*, *ampH*, *mecB*, *Gob-12*, *mecD*, *ampC1*, *NmcR*
	Cefuroxime	R	

Aminoglycosides	—	—	*smeR*, *cpxA*, *baeR*, *baeS*, *smeS*, *kdpE*, *aadA7*, *cpxA*, *KpnE*, *mecD*, *OmpK37*, *ampC1*, *NmcR*
		—	

Macrolides	—	—	*evgS*, *oleB*, *macB*, *efrA*, *CRP*, *optrA*, *mtrA*, *Erm*(*34*), *tlrC*, *evgA*, *oleC*, *lmrC*, *macA*, *KpnE*, *AmvA*, *emrE*, *H-NS*, *chrB*, *lsaC*, *oleB*, *mphB*, *carA*, *TolC*, *CpxR*

Tetracyclines	Doxycycline	R	*evgS*, *oleB*, *adeL*, *tetA*(*58*), *optrA*, *tetB*(*P*), *tetW*, *ramA*, *soxR*, *tetS*, *marA*, *tlrC*, *tetB*(*60*), *acrB*, *acrA*, *AcrS*, *optrA*, *evgA*, *lmrC*, *mdfA*, *evgS*, *adeG*, *KpnE*, *KpnF*, *marA*, *adeL*, *H-NS*, *tcr3*, *lsaC*, *sdiA*, *tetB*(*46*), *oqxA*, *CpxR*, *otr*(*A*)
	Tetracycline	R	

Quinolones	Levofloxacin	R	*evgS*, *adeL*, *efrA*, *CRP*, *ramA*, *soxR*, *gadX*, *qacH*, *mdtM*, *marA*, *patA*, *acrB*, *acrA*, *acrS*, *evgA*, *patB*, *arlR*, *abaQ*, *mdtH*, *hmrM*, *adeG*, *adeL*, *H-NS*, *sdiA*, *mdtK*, *lfrA*, *emrR*, *emrA*, *emrB*, *TolC*, *oqxA*, *cpxR*, *QepA2*
	Norfloxacin	R	
	Ciprofloxacin	R	

Sulfonamides	Sulfamethoxazole	R	*sul4*, *CpxR*

Lincosamides	—	—	*oleB*, *lmrD*, *optrA*, *mdtM*, *Erm*(*34*), *tlrC*, *lmrC*, *lmrD*, *chrB*, *lsaC*, *cfrA*, *carA*

Ansamycins	—	—	*efrA*, *ramA*, *soxR*, *marA*, *acrB*, *acrA*, *AcrS*, *rphB*, *KpnE*, *sdiA*, *TolC*, *rpoB2*, *efpA*

## Data Availability

The data that support the findings of this study are available from the corresponding author, Yan Su (2006au@163.com), upon reasonable request. Sequencing data for the *S*. Abortusequi isolates XJP1 and XJS1, as well as the associated plasmid, have been deposited in GenBank under the accession numbers CP161946 (XJP1), CP170123 (XJS1), and CP161947 (plasmid).

## Supporting Information

Supporting Information Additional supporting information includes The ARRIVE guidelines 2.0. checklist.

## References

[1] Lokken K. L., Walker G. T., Tsolis R. M. (2016). Disseminated Infections With Antibiotic-Resistant Non-Typhoidal *Salmonella* Strains: Contributions of Host and Pathogen Factors. *Pathogens and Disease*.

[2] White A. E., Tillman A. R., Hedberg C. (2022). Foodborne Illness Outbreaks Reported to National Surveillance, United States, 2009–2018. *Emerging Microbes & Infections*.

[3] Wang Z., Huang C. H., Liu Y. H. (2024). *Salmonellosis* Outbreak Archive in China: Data Collection and Assembly. *Scientific Data*.

[4] Issenhuth-Jeanjean S., Roggentin P., Mikoleit M. (2014). Supplement 2008–2010 (no. 48) to the White–Kauffmann–Le Minor Scheme. *Research in Microbiology*.

[5] Han J., Aljahdali N., Zhao S. (2024). Infection Biology of *Salmonella enterica*. *EcoSal Plus*.

[6] Grandolfo E., Parisi A., Ricci A. (2018). High Mortality in Foals Associated With *Salmonella enterica* Subsp. *enterica* Abortusequi Infection in Italy. *Journal of Veterinary Diagnostic Investigation*.

[7] Di Gennaro E. E., Guida N., Franco P. G., Moras E. V., Munoz A. J. (2012). Infectious Abortion Caused by *Salmonella enterica* Subsp Enterica Serovar Abortusequi in Argentina. *Journal of Equine Veterinary Science*.

[8] Stritof Z., Habus J., Grizelj J. (2016). Two Outbreaks of Salmonella Abortusequi Abortion in Mares in Croatia. *Journal of Equine Veterinary Science*.

[9] Chandra M., Singh B. R., Babu N., Ravi K. A., Mahtab Z. S. (2014). Anti-Abortion and Fertility Vaccine Potential of Defined Double Deletion(ΔaroAΔhtrA) Mutant (S30) of, *Salmonella*, Abortusequi in Equids. *Journal of Equine Veterinary Science*.

[10] Neustroev M. P., Petrova S. G. (2020). Developmental Results of a Vaccine Against *Salmonella*-Induced Equine Abortion. *Russian Agricultural Sciences*.

[11] Niwa H., Hobo S., Kinoshita Y. (2016). Aneurysm of the Cranial Mesenteric Artery as a Site of Carriage of *Salmonella enterica* Subsp. Enterica Serovar Abortusequi in the Horse. *Journal of Veterinary Diagnostic Investigation*.

[12] Wang H., Liu K. J., Sun Y. H. (2019). Abortion in Donkeys Associated With *Salmonella* abortus equi Infection. *Equine Veterinary Journal*.

[13] Juffo G. D., Bassuino D. M., Gomes D. C. (2017). Equine salmonellosis in Southern Brazil. *Tropical Animal Health and Production*.

[14] Marenzoni M. L., Lepri E., Casagrande Proietti P. (2012). Causes of Equine Abortion, Stillbirth and Neonatal Death in Central Italy. *Veterinary Record*.

[15] Bustos C. P., Moroni M., Caffer M. I. (2020). Genotypic Diversity of *Salmonella* ser. Abortusequi Isolates From Argentina. *Equine Veterinary Journal*.

[16] Bukhari S. S. U. H., Rosanowski S. M., McElligott A. G., Parkes R. S. V. (2022). Welfare Concerns for Mounted Load Carrying by Working Donkeys in Pakistan. *Frontiers in Veterinary Science*.

[17] Zhu M., Liu W., Zhang L. (2021). Characterization of *Salmonella* Isolated From Donkeys During an Abortion Storm in China. *Microbial Pathogenesis*.

[18] Wu Y., Jiang T., Bao D. (2023). Global Population Structure and Genomic Surveillance Framework of Carbapenem-Resistant *Salmonella enterica*. *Drug Resistance Updates*.

[19] Zhu X., Zhao Z., Ma S. (2020). *Brucella melitensis*, a Latent “Travel Bacterium,” Continual Spread and Expansion From Northern to Southern China and Its Relationship to Worldwide Lineages. *Emerging Microbes & Infections*.

[20] Sacchini L., Wahab T., Di Giannatale E. (2019). Whole Genome Sequencing for Tracing Geographical Origin of Imported Cases of Human Brucellosis in Sweden. *Microorganisms*.

[21] Zakaria Z., Hassan L., Sharif Z. (2020). Analysis of *Salmonella enterica* Serovar Enteritidis Isolates From Chickens and Chicken Meat Products in Malaysia Using PFGE, and MLST. *BMC Veterinary Research*.

[22] Nguyen L. T., Schmidt H. A., von Haeseler A., Minh B. Q. (2015). IQ-TREE: A Fast and Effective Stochastic Algorithm for Estimating Maximum-Likelihood Phylogenies. *Molecular Biology and Evolution*.

[23] Naidoo N., Zishiri O. T. (2023). Comparative Genomics Analysis and Characterization of Shiga Toxin-Producing, *Escherichia coli*, O157: H7 Strains Reveal Virulence Genes, Resistance Genes, Prophages and Plasmids. *BMC Genomics*.

[24] Toyting J., Nuanmuang N., Utrarachkij F. (2024). Genomic Analysis of *Salmonella* Isolated From Canal Water in Bangkok, Thailand. *Microbiology Spectrum*.

[25] Alikhan N. F., Petty N. K., Ben Zakour N. L., Beatson S. A. (2011). BLAST Ring Image Generator (BRIG): Simple Prokaryote Genome Comparisons. *BMC Genomics*.

[26] Liu B., Zheng D., Zhou S., Chen L., Yang J. (2022). VFDB 2022: A General Classification Scheme for Bacterial Virulence Factors. *Nucleic Acids Research*.

[27] CLSI (2022). Performance Standards for Antimicrobial Susceptibility Testing.

[28] Ferrari R. G., Rosario D. K. A., Cunha-Neto A., Mano S. B., Figueiredo E. E. S., Conte-Junior C. A. (2019). Worldwide Epidemiology of *Salmonella* Serovars in Animal Based Foods: A Meta-Analysis. *Applied and Environmental Microbiology*.

[29] Wang Z., Zhou H., Liu Y. (2024). Nationwide Trends and Features of Human Salmonellosis Outbreaks in China. *Emerging Microbes & Infections*.

[30] Chen J., Huang L., An H. (2024). One Health Approach Probes Zoonotic Non-Typhoidal *Salmonella* Infections in China: A Systematic Review and Meta-Analysis. *Journal of Global Health*.

[31] Wang Z., Zhou H., Liu Y. (1993). Equine Abortion and Stillbirth in Central Kentucky During 1988 and 1989 Foaling Seasons. *Journal of Veterinary Diagnostic Investigation*.

[32] Spier S. J. (1993). Salmonellosis. *Veterinary Clinics of North America-Equine Practice*.

[33] Akiba M., Uchida I., Nishimori K. (2003). Comparison of *Salmonella enterica* Serovar Abortusequi Isolates of Equine Origin by Pulsed-Field Gelelectrophoresis and Fluorescent Amplified-Fragment Length Polymorphism Fingerprinting. *Veterinary Microbiology*.

[34] Jiang Z., Li D., Liu Z. (2023). Genomic Typing and Virulence Gene Profile Analysis of Salmonella Derby From Different Sources. *Microbial Pathogenesis*.

[35] Tiengo A., Orsini M., Petrin S. (2023). Whole-Genome Sequence of *salmonella enterica* Serovar Bispebjerg From Turkey Reveals Its Pathogenic Potential. *Microbiology Resource Announcements*.

[36] Traglia G. M., Betancor L., Yim L., Iriarte A., Chabalgoity J. A. (2024). Genotypic and Phenotypic Analysis of *Salmonella enterica* Serovar Derby, Looking for Clues Explaining the Impairment of Egg Isolates to Cause Human Disease. *Frontiers in Microbiology*.

[37] Alvarez D. M., Barrón-Montenegro R., Conejeros J., Rivera D., Undurraga E. A., Moreno-Switt A. I. (2023). A Review of the Global Emergence of Multidrug-Resistant *Salmonella* Enterica Subsp. Enterica Serovar Infantis. *International Journal of Food Microbiology*.

[38] Antunes P., Mourão J., Campos J., Peixe L. (2016). *Salmonellosis*: The Role of Poultry Meat. *Clinical Microbiology and Infection*.

[39] Pribul B. R., Festivo M. L., Rodrigues M. S. (2017). Characteristics of Quinolone Resistance in *Salmonella* spp. Isolates From the Food Chain in Brazil. *Frontiers in Microbiology*.

[40] AbuOun M., Jones H., Stubberfield E. (2021). On Behalf of the Rehab Consortium. A Genomic Epidemiological Study Shows That Prevalence of Antimicrobial Resistance in *Enterobacterales* Is Associated With the Livestock Host, as Well as Antimicrobial Usage. *Microbial Genomics*.

[41] Pan H., Zhou X., Chai W. (2019). Diversified Sources for Human Infections by *Salmonella enterica* Serovar Newport. *Transboundary and Emerging Diseases*.

[42] Hou Z., Xu B., Liu L., Yan R., Zhang J. (2024). Isolation, Identification, Antimicrobial Resistance, Genotyping, and Whole-Genome Sequencing Analysis of Salmonella Enteritidis Isolated From a Food-Poisoning Incident. *Polish Journal of Microbiology*.

[43] Belloy L., Decrausaz L., Boujon P., Hächler H., Waldvogel A. S. (2009). Diagnosis by Culture and PCR of, *Salmonella*, Abortusovis Infection Under Clinical Conditions in Aborting Sheep in Switzerland. *Veterinary Microbiology*.

[44] Payne M., Williamson S., Wang Q. (2024). Emergence of Poultry-Associated Human *Salmonella enterica* Serovar Abortusovis Infections, New South Wales, Australia. *Emerging Infectious Diseases*.

[45] Guerra B., Soto S., Helmuth R., Mendoza M. C. (2002). Characterization of a Self-Transferable Plasmid From *Salmonella enterica* Serotype Typhimurium Clinical Isolates Carrying Two Integron-Borne Gene Cassettes Together With Virulence and Drug Resistance Genes. *Antimicrobial Agents and Chemotherapy*.

[46] Felix M.A., Sopovski D., Commichaux S. (2024). Genetic Relatedness and Virulence Potential of *Salmonella* Schwarzengrund Strains With or Without an IncFIB-IncFIC (FII) Fusion Plasmid Isolated From Food and Clinical Sources. *Frontiers in Microbiology*.

[47] Mansour M. N., Yaghi J., Khoury A. E. (2020). Prediction of *Salmonella* Serovars Isolated From Clinical and Food Matrices in Lebanon and Genomic-Based Investigation Focusing on Enteritidis Serovar. *International Journal of Food Microbiology*.

[48] Wu S. Y., Wang L. D., Li J. L. (2016). *Salmonella Spv* Locus Suppresses Host Innate Immune Responses to Bacterial Infection. *Fish & Shellfish Immunology*.

[49] Passaris I., Cambre A., Govers S. K., Aertsen A. (2018). Bimodal Expression of the, *Salmonella*, Typhimurium *spv* Operon. *Genetics*.

[50] Uzzau S., Gulig P. A., Paglietti B., Leori G., Stocker B. A., Rubino S. (2000). Role of the, *Salmonella abortusovis* Virulence Plasmid in the Infection of BALB/c Mice. *FEMS Microbiology Letters*.

[51] Sun L., Yang S., Deng Q. (2020). *Salmonella* Effector SpvB Disrupts Intestinal Epithelial Barrier Integrity for Bacterial Translocation. *Frontiers in Cellular and Infection Microbiology*.

[52] Zhou L., Li Y., Gao S. (2021). *Salmonella spvC* Gene Inhibits Autophagy of Host Cells and Suppresses NLRP3 as Well as NLRC4. *Frontiers in Immunology*.

[53] Khan M. M., Ali A., Kolenda R. (2023). The Role of AJB35136 and fdtA Genes in Biofilm Formation by Avian Pathogenic *Escherichia coli*. *BMC Veterinary Research*.

[54] Seo S. W., Kim D., O’Brien E. J., Szubin R., Palsson B. O. (2015). Decoding Genome-Wide GadEWX-Transcriptional Regulatory Networks Reveals Multifaceted Cellular Responses to Acid Stress in *Escherichia coli*. *Nature Communications*.

[55] Shin S., Castanie-Cornet M. P., Foster J. W., Crawford J. A., Brinkley C., Kaper J. B. (2001). An Activator of Glutamate Decarboxylase Genes Regulates the Expression of Enteropathogenic *Escherichia coli* Virulence Genes Through Control of the Plasmid-Encoded Regulator, Per. *Molecular Microbiology*.

[56] Branchu P., Matrat S., Vareille M. (2014). NsrR, GadE, and GadX Interplay in Repressing Expression of the, *Escherichia coli*, O157:H7 LEE Pathogenicity Island in Response to Nitric Oxide. *PLoS Pathogens*.

[57] Braun H. S., Sponder G., Aschenbach J. R., Kerner K., Bauerfeind R., Deiner C. (2017). The GadX Regulon Affects Virulence Gene Expression and Adhesion of Porcine Enteropathogenic *Escherichia coli* in Vitro. *Veterinary and Animal Science*.

[58] Schwan W. R., Luedtke J., Engelbrecht K. (2022). Regulation of *Escherichia coli* Fim Gene Transcription by GadE and Other Acid Tolerance Gene Products. *Microbiology*.

[59] Tran-Dien A., Le Hello S., Bouchier C., Weill F. X. (2018). Early Transmissible Ampicillin Resistance in Zoonotic *Salmonella enterica* Serotype Typhimurium in the Late 1950s: A Retrospective, Whole-Genome Sequencing Study. *The Lancet Infectious Diseases*.

[60] Randall L. P., Cooles S. W., Coldham N. C., Stapleton K. S., Piddock L. J., Woodward M. J. (2006). Modification of Enrofloxacin Treatment Regimens for Poultry Experimentally Infected With *Salmonella enterica* Serovar Typhimurium DT104 to Minimize Selection of Resistance. *Antimicrobial Agents and Chemotherapy*.

[61] Kang J., Hossain M. A., Park H. C., Kim Y., Lee K. J., Park S. W. (2019). Pharmacokinetic and Pharmacodynamic Integration of Enrofloxacin Against *Salmonella* Enteritidis After Administering to Broiler Chicken by Per-Oral and Intravenous Routes. *Journal of Veterinary Science*.

[62] Shang K., Wei B., Kang M. (2018). Distribution and Dissemination of Antimicrobial-Resistant *Salmonella* in Broiler Farms With or Without Enrofloxacin Use. *BMC Veterinary Research*.

[63] Cuypers W. L., Jacobs J., Wong V., Klemm E. J., Deborggraeve S., Van Puyvelde S. (2018). Fluoroquinolone Resistance in *Salmonella*: Insights by Whole-Genome Sequencing. *Microbial Genomics*.

